# Pediatric *delirium* in times of COVID-19

**DOI:** 10.5935/0103-507X.20210070

**Published:** 2021

**Authors:** Roberta Esteves Vieira de Castro, Miguel Rodríguez-Rubio, Maria Clara de Magalhães-Barbosa, Arnaldo Prata-Barbosa

**Affiliations:** 1 Universidade do Estado do Rio de Janeiro - Rio de Janeiro (RJ), Brasil.; 2 Instituto D’Or de Pesquisa e Ensino - Rio de Janeiro (RJ), Brasil.; 3 Department of Pediatric Intensive Care, Hospital Universitário La Paz, Faculdad de Medicina, Universidad Autónoma de Madrid - Madrid, Spain.

## Introduction

*Delirium* is defined as a neurocognitive syndrome characterized by the acute onset of brain dysfunction with fluctuations in the basal mental state, inattention and disorganized thinking or altered levels of consciousness.^(^1^,^2^)^ It is a frequent complication in intensive care units (ICUs).^(^3^)^ Its occurrence is strongly predictive of an increase in the duration of mechanical ventilation (MV), length of stay in the ICU and hospital, risk of falls, hospital costs and mortality.^(^4^)^

Data from the literature show that the exact incidence of *delirium* in pediatric patients is still unknown and ranges from 0.84% to 66%. The prevalence is also quite variable at between 13% and 66% and has been well characterized since the implementation of valid screening tools for *deliriu*m in pediatric populations. Higher prevalence values of between 50% and approximately 70% were reported in critically ill children under 5 years of age, those on MV, during the postoperative period of cardiac surgery and during the immediate postoperative period after general anesthesia and elective surgery. However, underdiagnosis or incorrect diagnosis of *delirium* may occur when it is based only on the clinical experience of the team, without the use of a valid and reliable tool.^(^2^,^5^,^6^)^

Considering the high prevalence of *delirium* in the ICU, it is believed to be affect at least one quarter of patients aged 65 years or older infected by severe acute respiratory syndrome coronavirus 2 (SARS-CoV-2) and more than two-thirds of patients with the most severe cases of coronavirus disease 2019 (COVID-19).^(^7^)^ However, *delirium* occurs not only in adults and the elderly but also in children and adolescents and has been associated with a wide spectrum of deleterious outcomes at all ages.^(^8^)^

Although the number of pediatric patients affected by COVID-19 is small compared to the number of affected adult and geriatric patients, children are also at risk of becoming seriously ill, especially in the presence of underlying diseases.^(^8^)^ Numerous studies have described these patients as less vulnerable and more predisposed to mild COVID-19. However, even with recent data on the reduced lethality of COVID-19 in pediatric patients in 2021 compared to 2020, children and adolescents may develop severe conditions, such as acute respiratory distress syndrome or multisystemic inflammatory syndrome.^(^9^-^11^)^

Although there are still many difficulties in determining the best COVID-19 management strategy, the need to maintain good clinical management practices is undeniable, especially during the pandemic.^(^12^-^14^)^ In this context, the occurrence of *delirium* in critically ill children with COVID-19 must be monitored. *Delirium* is a frequent manifestation in infectious conditions and can be considered an early marker of acute disease, in addition to being associated with long-term cognitive complications.^(^13^,^15^)^ Due to the development of valid and reliable tools for the diagnosis of *delirium* in pediatrics, it is now known that one in four children hospitalized in the ICU is likely to present it.^(^5^)^ Its prevalence may be higher in special subgroups, such as patients undergoing cardiac surgery or extracorporeal membrane oxygenation.^(^5^,^16^)^ The recognition of *delirium* in pediatrics is extremely relevant because it has been independently associated with increased costs in the pediatric ICU, prolonged hospital stays and increased in-hospital mortality.^(^5^,^17^,^18^)^

### Risk factors for delirium in patients with COVID-19

Often, *delirium* is triggered by more than one risk factor. The probability of its occurrence increases with the increase in the number of these factors, and an understanding of this association is essential for identifying potentially reversible causes. *Delirium* emerges as the result of an intricate relationship between vulnerability factors and precipitating factors, i.e., a patient with high vulnerability may develop it in the presence of a minor injury, while one with few predisposing factors may require more intense injuries to develop it.^(^19^)^

Important factors that predispose pediatric patients to the development of *delirium* include age less than 2 years and a history of neurodevelopmental delay. Patients with immature or abnormal brains are more prone to developing *delirium*, as are elderly individuals and patients with underlying dementia.^(^20^)^ Other predisposing factors are previous comorbidities, the severity of the underlying disease, malnutrition (associated with a serum albumin level below 3.0g/dL) and MV dependence. Predisposing factors are inherent to the patient and cannot be modified. The most frequent precipitating factors in pediatrics include the use of benzodiazepines and anticholinergic drugs, cardiac bypass surgeries, bed immobilization, prolonged hospitalization in the pediatric ICU, use of physical restraints, pain and withdrawal syndrome. These factors act as triggers and can be modified by the health team in many cases.^(^20^,^21^)^

The development of *delirium* is closely related to the severity of the disease. In the context of SARS-CoV-2 infection, recent studies have shown that *delirium* may be triggered by factors such as hypoxia and the resulting deficiency in cerebral oxygenation; neuronal inflammation due to the cytokine storm resulting from an unbalanced immune system activation; and/or direct invasion of the central nervous system by the virus, which has neuronal toxicity.^(^15^)^ In addition to factors associated with COVID-19, such as neuroinflammation, multiple organ failure and increased risk of thrombosis, treatment-related factors may increase the risk of *delirium*. These factors include the use of prolonged MV with deep sedation and the iatrogenic environment of the pediatric ICU, which is marked by intense sleep deprivation.^(^8^,^22^)^ In addition to these factors, there is the need for isolation imposed by COVID-19 to reduce the exposure of health professionals, which decreases contact with the team and may be aggravated by the scarcity of personal protective equipment (PPE). Although this isolation is understandable given the intensity of the pandemic, this reality increases patients’ isolation and immobility and, when associated with the numerous complications of the disease, produces an extremely iatrogenic environment with a high risk of *delirium*.^(^23^)^

### Strategies for the prevention and management of delirium in pediatric ICUs during the COVID-19 pandemic

The measures adopted to prevent the spread of SARS-CoV-2, such as the use of PPE and restrictive visitation policies, in addition to the scarcity of professionals available for care (which reduces the time available for evaluations), may hinder the recognition of *delirium* and create barriers for the implementation of recommended nonpharmacological strategies. In addition, these measures may impair patient orientation and are a significant risk factor for the development of *delirium*.^(^22^)^

Although extremely relevant, *delirium* is often unrecognized, and the pandemic has presented numerous obstacles to its diagnosis.^(^12^,^22^,^23^)^ A team effort is required to adopt strategies that reduce these barriers. One of the most efficient resources is the adequate use of validated tools for screening for *delirium* in critically ill children.^(^8^)^ The diagnostic criteria for *delirium* represent a valid and operationalized construct with high reliability and remarkable clinical application. The use of homogeneous and validated nomenclature can help the team avoid vague terms, such as “altered mental state”, thus enabling the incorporation of standardized strategies for the management of *delirium* while facilitating communication with patients, family members and among themselves and other health professionals.^(^7^)^

Table 1 presents some proposed measures for the prevention and management of *delirium* in pediatric patients that have been adapted to the context of the pandemic. These measures do not require the implementation of complex actions and do not increase the risk of exposure for health professionals. It is believed that, as with any serious childhood disorder, the prevention, evaluation and treatment of *delirium* should be part of the approach taken for pediatric patients with COVID-19. Investing time in this approach can avoid costs and associated complications.

**Table 1:** Recommendations for the diagnosis, prevention and treatment of *delirium* in pediatric patients with COVID-19

Diagnosis^(^12^,^21^,^22^,^24^)^	Evaluate the patient’s baseline mental state, obtaining information from a reliable informant. For pediatric patients, it is recommended that a single family member or other companion authorized by the parents remain with the child at all times. The companion must follow the norms of the unit and obey the recommended isolation measures (surgical mask, apron and frequent hand washing). Ideally, the companion should always be the same person. In the absence of a companion for the child, information about the child’s basic mental state can be obtained by telephone interview Use of a valid and reliable tool: psCAM-ICU, pCAM-ICU, CAPD or SOS-PD*. The tool should be administered at some point during every 12-h shift
Identification of risk factors^(^3^,^21^,^22^)^	Identify and address risk factors, including pain and withdrawal syndrome Pay attention to bladder, fecaloma, hypoxemia, overlapping infections, dehydration, electrolyte disorders and polypharmacy (review prescriptions)
Nonpharmacological measures^(^1^,^25^-^31^)^	Consider environmental changes: provide a calm and peaceful environment that is consistent and predictable; consider moving patients with hyperactive or mixed *delirium* to a bed in a quieter location and moving patients with hypoactive *delirium* to a bed in a location with greater interaction; verify the possibility of letting the child have an object that is familiar to him or her; use physical restraints as a last resort; provide glasses or hearing aids to children who use these devices; explore the use of electronic devices (smartphones or tablets) for communication with the family if the child is alone Adopt communication strategies: speak calmly and slowly using short and clear sentences, explaining to the child where he or she is and why he or she needs to stay there; identify oneself and describe what is being done; tell the child the time of day and day of the week; do not discuss visual or auditory hallucinations with child, and instead simply explain that their perceptions are different; when possible, talk to the child about real people and events Promote sleep: wake the child at the same time every morning; leave the bed in a chair-like position similar when possible according to the child’s age and tolerance; discourage daytime sleep, except for scheduled naps or periods of silent rest; use a weak night light to reduce the child’s misperceptions and fears at night; use masks to block light during sleep and earplugs or white noise for sound masking; avoid overstimulation, especially before scheduled sleep or rest times; try to concentrate team activities during the day to avoid sleep interruptions at night; make a calendar and clock available for identifying the date and time Encourage mobilization and cognitive stimulation activities: adopt consistent daily routines for hygiene, mobility, range of motion exercises, therapies, interventions and play Cluster care: concentrate interventions to be performed with the patient to minimize interruptions and noise during rest periods Behavioral therapies: directed relaxation techniques that use cognitive behavioral resources and can be applied by qualified professionals on the multidisciplinary team, such as psychological therapy, occupational therapy, music therapy, aromatherapy, pet therapy and play therapy Breastfeeding and non-nutritive sucking with oral solutions of sucrose and/or glucose in patients with an oral diet whose clinical condition allows it. These strategies can be used with neonates and infants undergoing mildly to moderately painful procedures alone or in combination with other pain relief strategies. Start 5 minutes before the painful procedure and, if possible, continue during the procedure Other non-pharmacological strategies, such as facilitated tucking (a technique that provides comfort and pain relief and that consists of keeping the extremities of the neonates or infants flexed and contained during a painful procedure), curling/swaddling (wrapping the body of the newborn or infant up to 6 months of age in a blanket/blankets, considering the clinical conditions, while keeping the arms close to the body to promote pain relief during painful procedures), and skin-to-skin contact and sensory stimulation (massage, caregiving) have been shown to be useful for reducing pain scores during short-term mildly to moderately painful procedures and should be used consistently

psCAM -ICU - Preschool Confusion Assessment Method for the Intensive Care Unit; pCAM-ICU - Pediatric Confusion Assessment Method for the Intensive Care Unit; CAPD - Cornell Assessment of Pediatric Delirium; SOS-PD - Sophia Observation Withdrawal Symptoms - Pediatric Delirium Scale.

* The pCAM-ICU tool has a version that has been translated and validated for Brazilian Portuguese. The CAPD and SOS-PD tools have only been translated (not yet validated).
